# Detection and classification of atrial and ventricular cardiovascular diseases to improve the cardiac health literacy for resource constrained regions

**DOI:** 10.1049/htl2.12043

**Published:** 2023-04-10

**Authors:** Neha Arora, Biswajit Mishra

**Affiliations:** ^1^ One Health Research Group DA‐IICT Gandhinagar India

**Keywords:** biomedical electronics, electrocardiography, signal classification

## Abstract

ECG is a non‐invasive way of determining cardiac health by measuring the electrical activity of the heart. A novel detection technique for feature points P, QRS and T is investigated to diagnose various atrial and ventricular cardiovascular anomalies with ECG signals for ambulatory monitoring. Before the system is worthy of field trials, it is validated with several databases and recorded their response. The QRS complex detection is based on the Pan Tompkins algorithm and difference operation method that provides positive predictivity, sensitivity and false detection rate of 99.29%, 99.49% and 1.29%, respectively. Proposed novel T wave detection provides sensitivity of 97.78%. Also, proposed P wave detection provides positive predictivity, sensitivity and false detection rate of 99.43%, 99.4% and 1.15% for the control study (normal subjects) and 82.68%, 94.3% and 25.4% for the case (patients with cardiac anomalies) study, respectively. Disease detection such as arrhythmia is based on standard R‐R intervals while myocardial infarction is based on the ST‐T deviations where the positive predictivity, sensitivity and accuracy are observed to be 94.6%, 84.2% and 85%, respectively. It should be noted that, since the frontal leads are only used, the anterior myocardial infarction cases are detected with the injury pattern in lead *avl* and ST depression in reciprocal leads. Detection of atrial fibrillation is done for both short and long duration signals using statistical methods using interquartile range and standard deviations, giving very high accuracy, 100% in most cases. The system hardware for obtaining the 2 lead ECG signal is designed using commercially available off the shelf components. Small field validation of the designed system is performed at a Public Health Centre in Gujarat, India with 42 patients (both cases and controls). 78.5% accuracy was achieved during the field validation. It is thus concluded that the proposed method is ideal for improvisation in cardiac health monitoring outreach in resource constrained regions.

## Graphical abstract

This paper demonstrates cardiovascular atrial and ventricular disease classification and the system implementation for the real‐time environment. Algorithms have been validated on the publicly available datasets before field validation of the system. Field validation of the designed system has been done in a Public Health Centre in India.

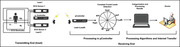

## 1 INTRODUCTION

Deaths due to cardiovascular diseases affect mostly low and middle income countries [1]. Timely detection can be beneficial towards primary health care if addressed. However, the early detection schemes in use require expensive and lengthy procedures in hospital settings and are not practical until serious symptoms appear. Therefore, a system that can detect cardiac anomalies at very early stages of the disease can be beneficial.

In literature, various methods are presented for R peak detection and classification. Gradl et al. [2] and Hadiyoso et al. [3] determine the R peaks of ECG QRS complex using the Pan Tompkins algorithm (PTA) [4, 5] and then detect only the rhythmic changes in R‐R interval. Padhy et al. [6], Lahiri et al. [7] and Weng et al. [8] detect and characterize MI with 12 lead ECG data available in the PTB database [9, 10] using neural networks and support vector machines. A 12 lead ECG along with 3 lead Vector Cardio Graph signals for Myocardial Infarction (MI) detection is reported by Correa et al. [11], where the access of 15 lead data in real‐time is challenging. Hernandez et al. [12] and Ross‐Howe et al. [13] use R peak and P wave information to determine only the atrial fibrillation. Jain et al. [14, 15] discuss PQRST point detection of ECG signals for identifying cardiovascular diseases using 12 leads signals. Although the methods provide better classification using Heart Rate Variability (HRV), it is challenging to implement these algorithms on portable devices used for ambulatory monitoring. In [14, 15], utilization of precordial leads requires expert supervision for placement of chest electrodes, posing limitations for ambulatory monitoring.

In this paper, we discuss an approach that requires the minimal intervention of a medical expert for the placement of electrodes, data acquisition and disease classification within home settings. Our contribution is summarized in the paper with extensive results and discussion. The proposed method provides findings and data that may indicate or assist the diagnosis of specific cardiac conditions. In this work, we obtain P, QRS and T points of ECG signals to categorize various atrial and ventricular anomalies of the heart. We propose a novel P and T detection method based on local search spaces, while for the QRS detection, the standard PTA and difference operation method is adopted. Noise effects are also discussed for the proposed P and T wave detection methods. This leads to efficient detection of HRV, arrhythmic conditions, atrial fibrillation, prolongation and shortening of QT and PR intervals and subsequent detection of MI. The use of 4 limb electrodes for obtaining the ECG signals offers ease of wearability which is one of the key contributions of this work for ambulatory monitoring [5, 16] applications. Additionally, user specific information is used along with the ECG data [17, 18] that improved detection accuracy.

## 2 ECG SYSTEM HARDWARE

**FIGURE 1:**
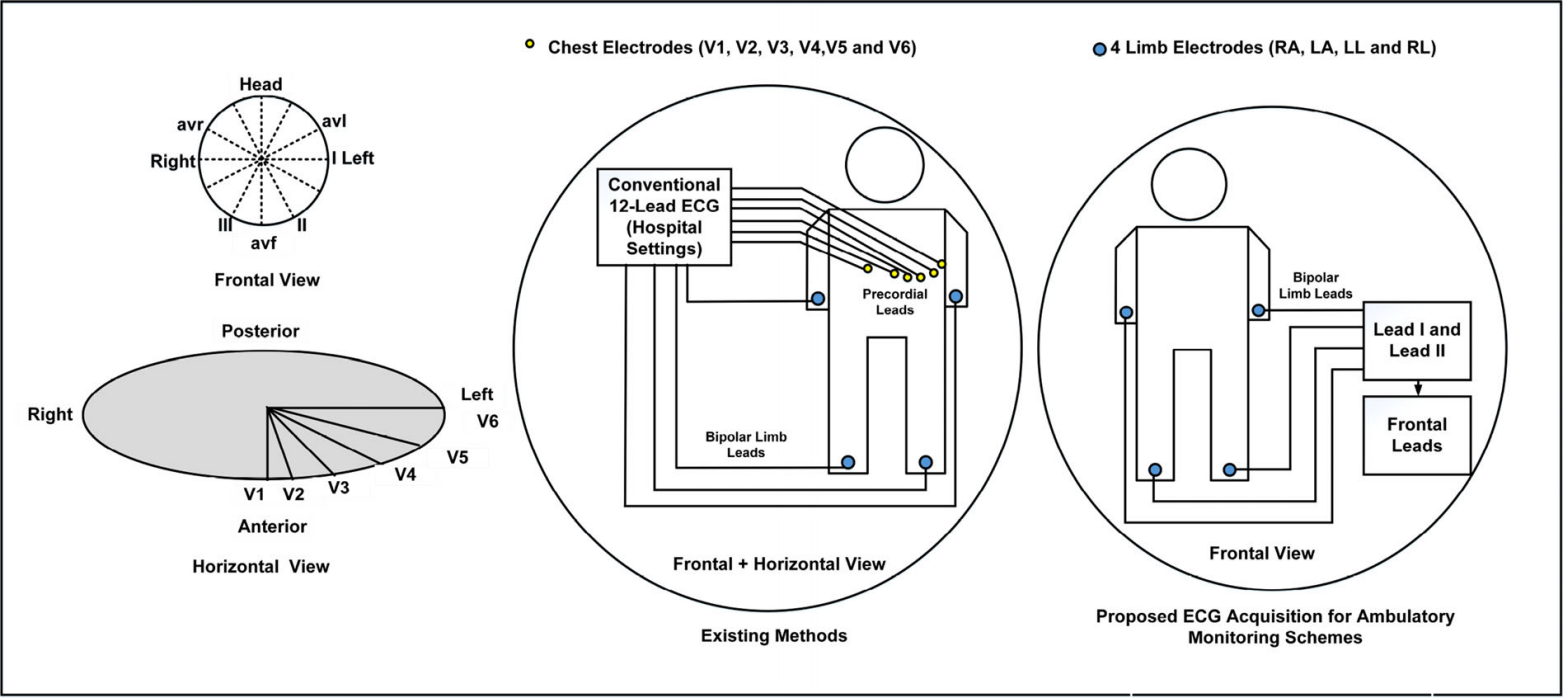
12 Lead versus proposed 2 lead electrodes configuration.

**FIGURE 2:**
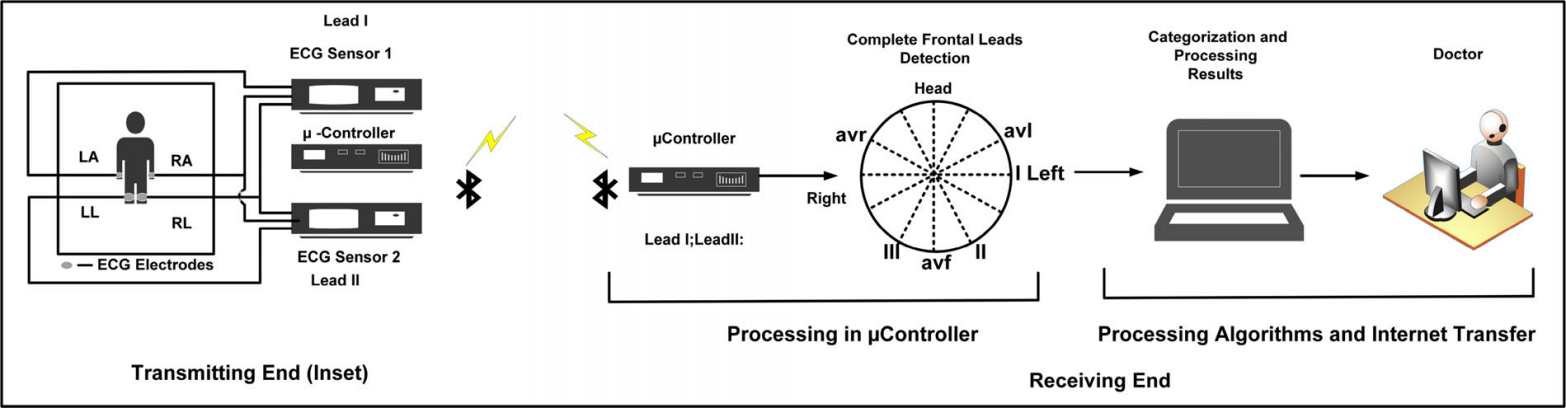
Proposed 2 lead system.

**FIGURE 3:**
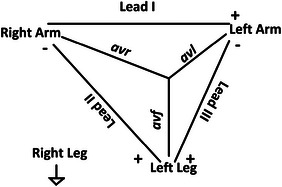
Einthoven's triangle and the augmented leads.

In a conventional 12 lead ECG system, 10 electrodes are connected to the body, that also requires expert supervision for electrode placement [16]. However, it is observed that in resource constrained regions in India and Africa, where the availability of doctors is severely limited, access to cardiac markers as primary screening remains a huge challenge [19]. To address this, several researches have been published where the focus shifts from hardware to software systems [20, 21]. We have addressed the affordability, automated detections and ease of use (with or without the medical personnel) as motivation for the system. As shown in Figure 1, our proposed system uses 4 electrodes that are connected to right arm (RA), left arm (LA), left leg (LL) and right leg (RL) to obtain lead I and lead II signals. Schematic of the proposed system is shown in Figure 2. The limb electrodes provide the complete frontal plane leads, while lead III and other augmented leads *avl, avf* and *avr* are derived from the Einthoven's Triangle [16] (Figure 3) with the following equations:

(1) LeadIII=LeadII−LeadI

(2) $$avl=\frac{Lead\;I-Lead\;III}{2}$$

(3) $$avf=\frac{Lead\;II+Lead\;III}{2}$$

(4) $$avr=\frac{-Lead\;I-Lead\;II}{2}$$

The augmented leads *avl, avr* and *avf* do not provide any extra details but are required for the frontal plane and is also useful for more accurate detections [16]. The schematic shown in Figure 2 is representing the developed system hardware which obtains lead I and lead II signals and transmits them to a nearby processor over a Bluetooth channel. We have designed the 2 lead front end system to obtain the lead I and lead II ECG signals for real‐time processing of algorithms.

**FIGURE 4:**
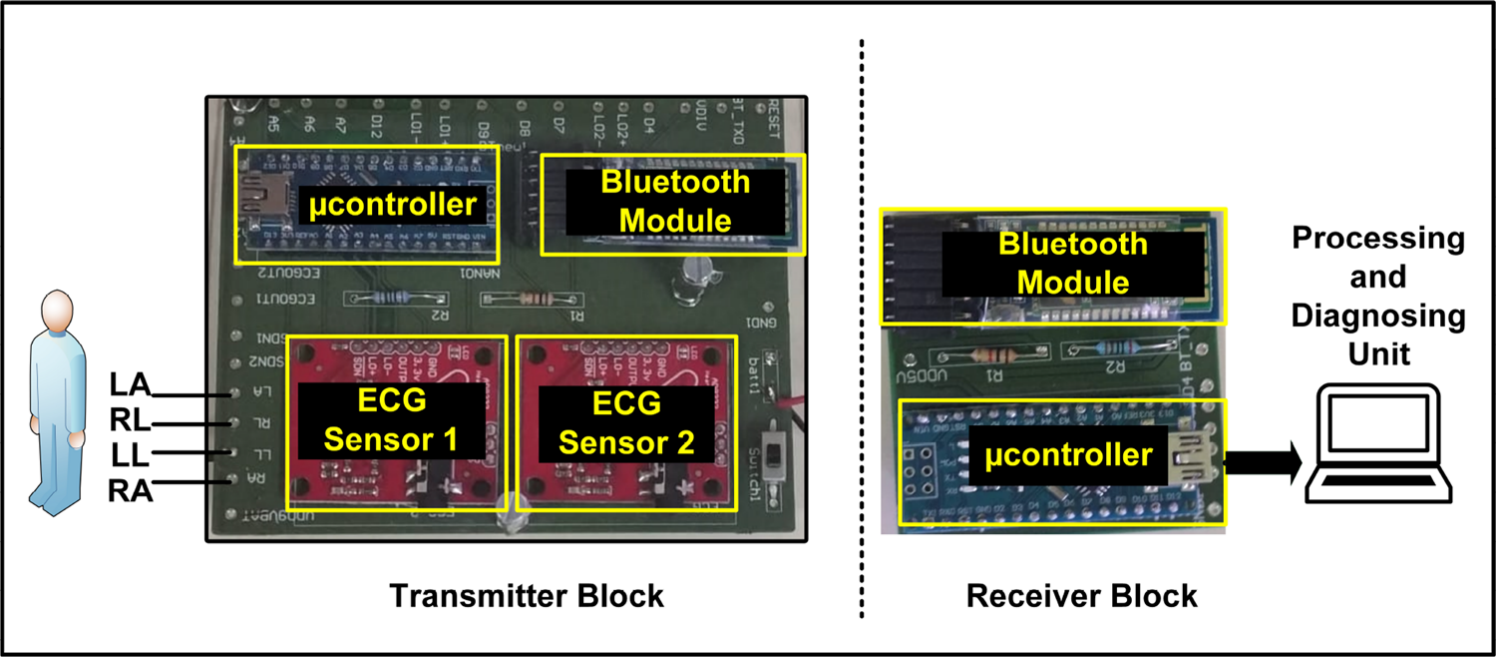
Real time implementation with the designed PCBs.

The front end and back end system diagrams of the PCBs are shown in Figure 4. At the receiver block, the signals are collected by another Bluetooth module where the algorithms for various cardiac conditions are processed in the local processor. It is worth noting that MI detection requires at least information from 6 frontal leads while other conditions require only the lead II signal [16].

**FIGURE 5:**
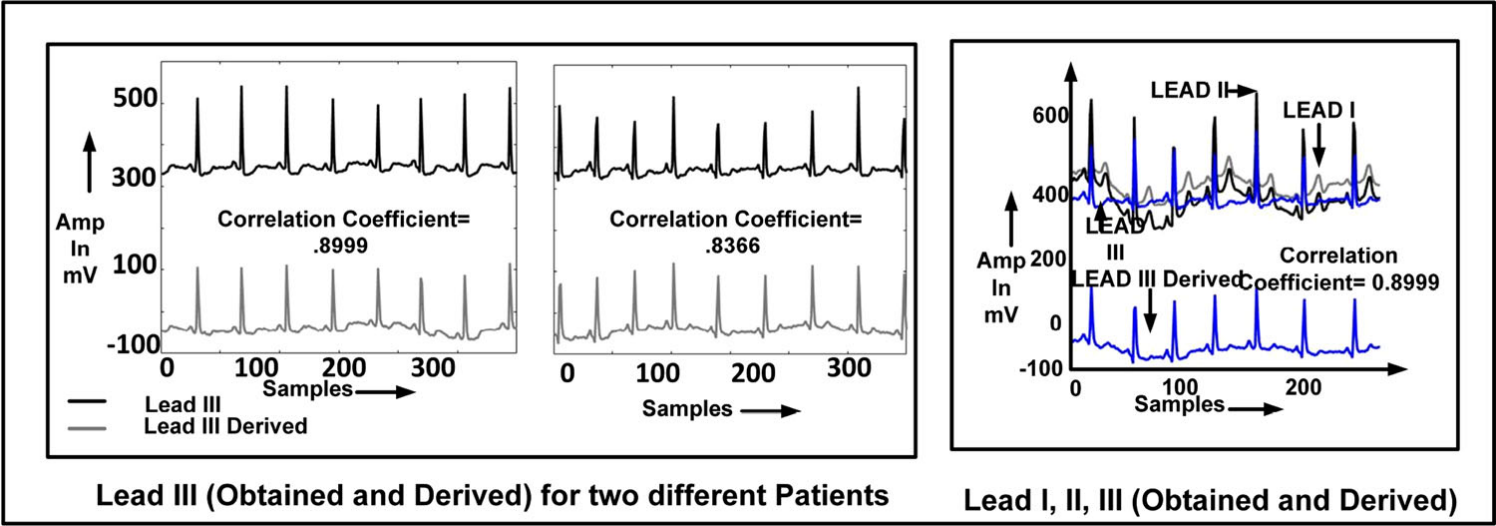
Real time implementation 2 lead front end results.

To verify the derived bipolar ECG signals, we initially acquired the three bipolar ECG signals (lead I, lead II, lead III), with three separate ECG acquisition boards. The obtained ECG lead III is compared with the derived lead III from Equation (1). It provided the correlation coefficient between the derived and obtained leads approximately close to 1, signifying the similarity of signals. Although the dc level of both signals is different, it does not cause any major implications on the obtained temporal morphological features. Hence, to save the area and power required by the third acquisition board, the system is optimized to have only two single lead ECG acquisition boards. Figure 5 shows the correlation coefficient between derived and obtained lead for two different cases and leads I, II and III signals. For all the experiments we have used a sampling frequency of 104Hz at a baud rate of 9600 for all the frontal leads, which is sufficient for ambulatory monitoring [22]. It is to be noted that for abnormal and normal signals of the PTB database, the correlation coefficient between the derived (lead III, *avl*, *avr* and *avf*) and existing leads (lead I, II) are perfectly unity.

## 3 FEATURE POINT DETECTION OF AN ECG SIGNAL

**FIGURE 6:**
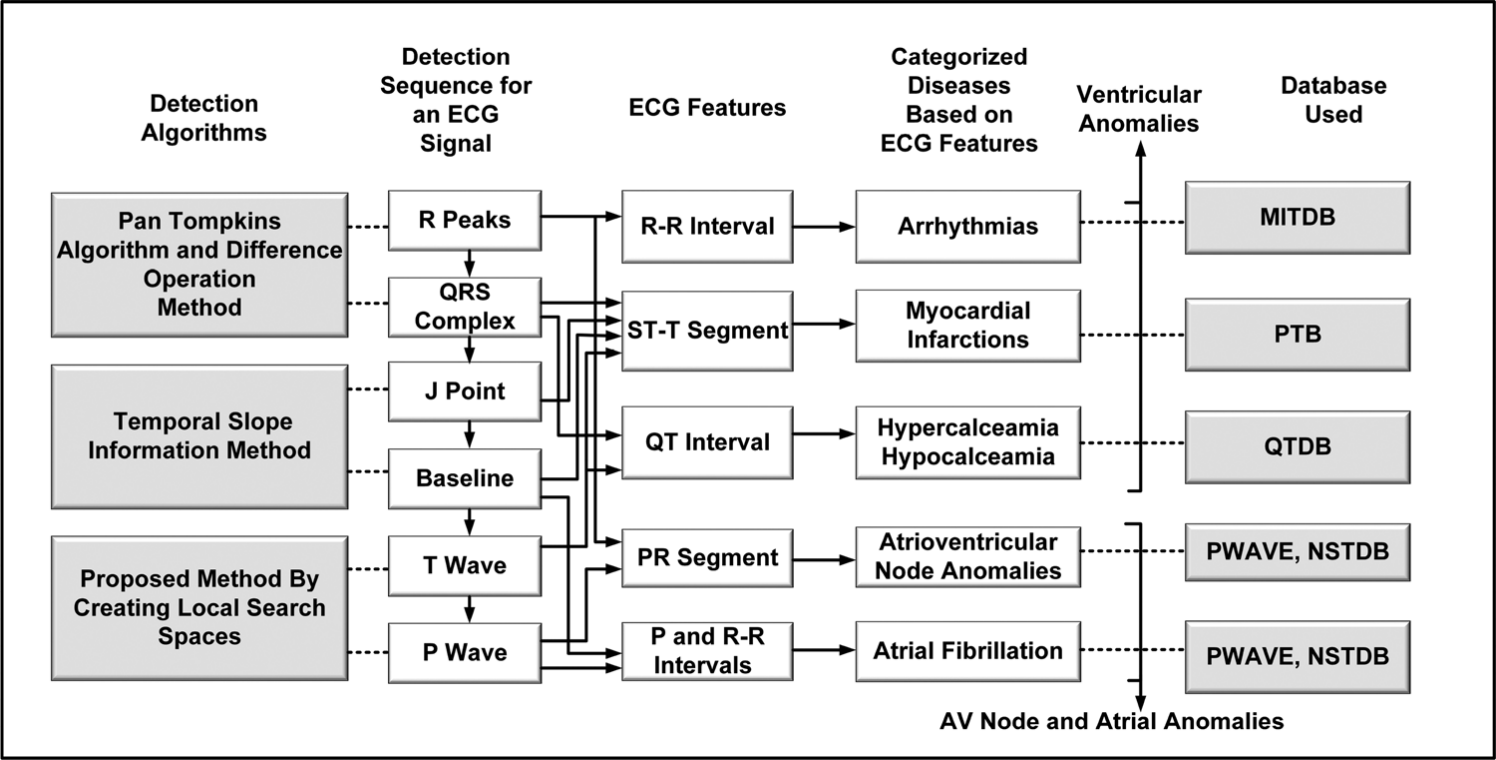
Detection sequence for an ECG signal and categorization.

**FIGURE 7:**
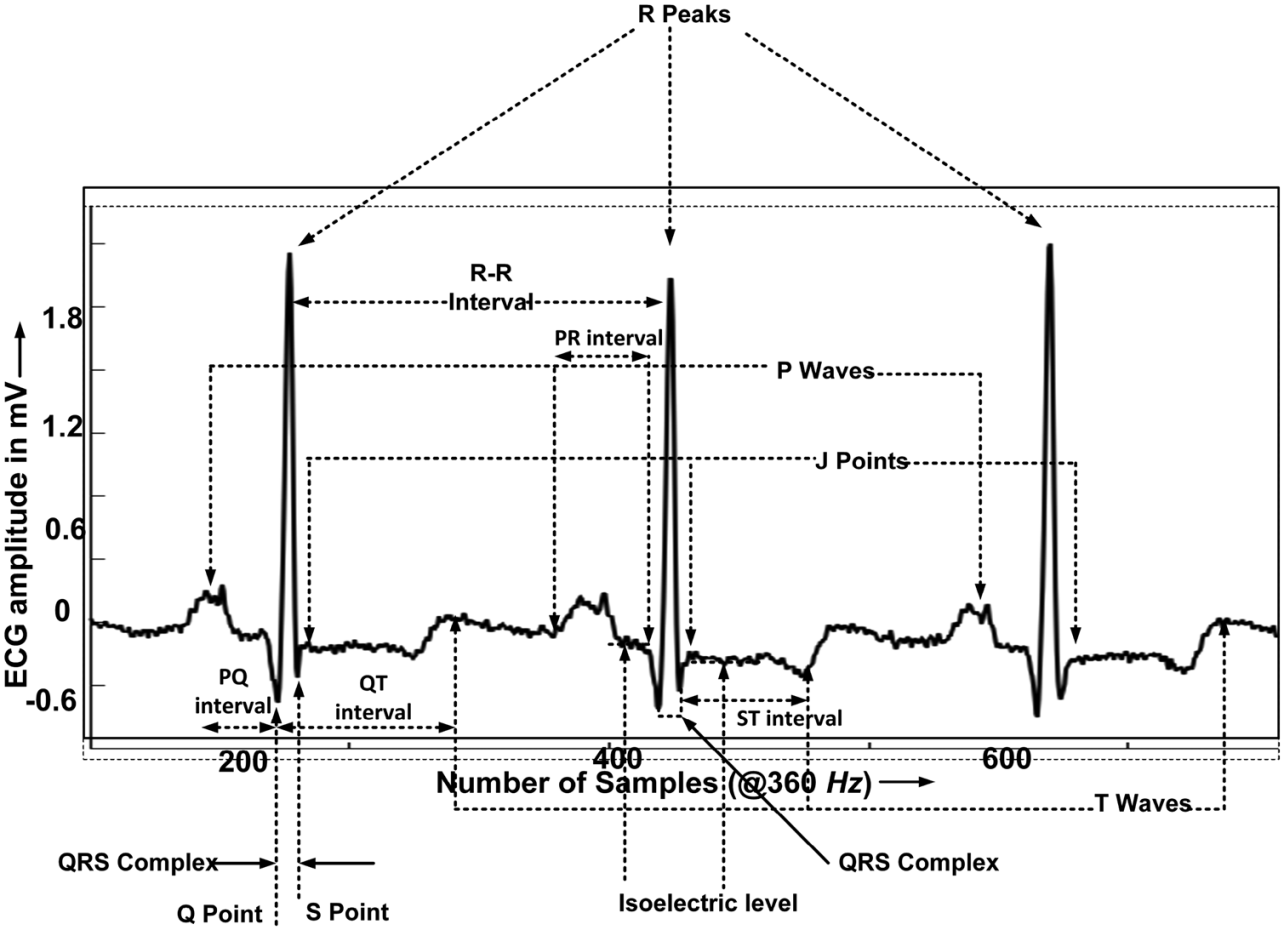
Various feature points in the ECG signal.

Categorization of several cardiac diseases requires the detection of feature points associated with ECG signal such as P, QRS, J, T and additional morphological changes. Our proposed method use a detection sequence shown in Figure 6. While, various feature points P, Q, R, S, T and J associated with ECG signals and various intervals RR, PR, QT, ST, PQ are shown in Figure 7 for categorization.

### 3.1 QRS complex detection

In our proposed method, initial step of the algorithm is to find the R peaks. For this, we have used the standard PTA [4, 5]. We also observed that the adaptive thresholding in PTA leads to a minimum acceptable false detection rate that can be useful for ambulatory monitoring schemes. In PTA, ECG signal is sent to a 5–15 Hz Band Pass Filter (BPF). The output of BPF is used for dual thresholding and is further passed through a five‐point differentiator. The signal that is normalized is then fed to a squaring block followed by Moving Average Integration Filter (MAF). MAF is specially designed to capture the widest QRS complex width (e.g. 150 ms in our case). As, the sampling frequency $\left(f_{s}\right)$ for the MIT‐BIH arrhythmia database [23] is 360 Hz, therefore we have chosen a 54 point $\left(f_{s}\times 150\;\text{ms}\right)$ MAF to capture QRS complexes. The MAF output along with the BPF output is used for adaptive thresholding to detect the R peaks in the signal. In this method, if the R peak is detected from both BPF and MAF, then that R peak is considered as the valid R peak.

**FIGURE 8:**
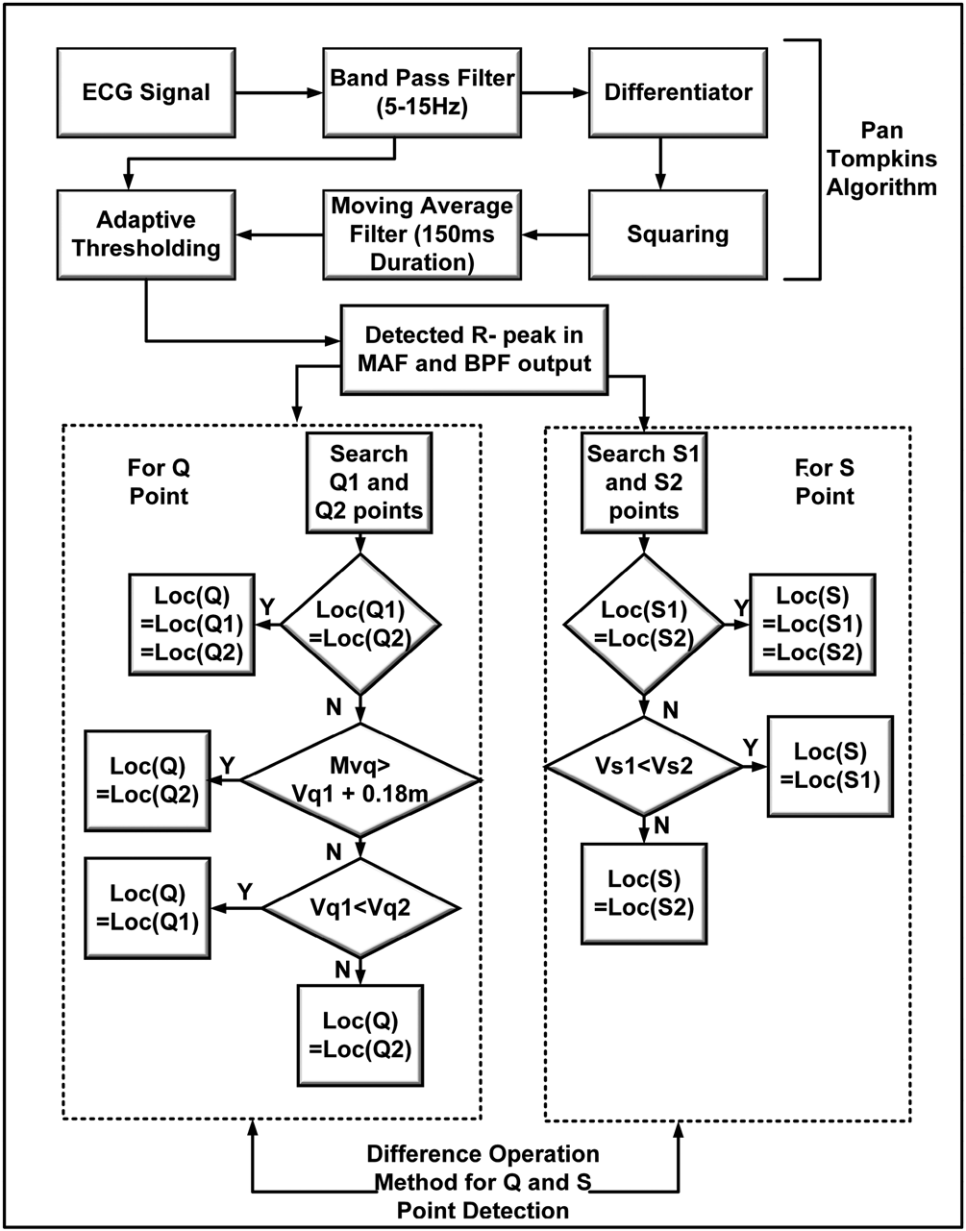
QRS detection of an ECG signal.

After determining the R peaks, we obtain the Q and S points to get complete information of QRS complex. Flow diagram shown in Figure 8 is based on the difference operation method [24] to determine the Q and S points locations and PTA for R peaks detections. To locate the Q point, two search spaces for Q1 and Q2 have been created adjacent to the R peak that are of 55.5 ms and 110 ms durations, respectively. The minimum point in the first search space has been marked as Q1 while the minimum point in the second duration is marked as Q2. If the location of Q1 and Q2 are identical, then the location is the actual Q point. If the maximum amplitude between Q2 and Q1 (termed as Mvq) is found to be greater than the sum of amplitude of Q1 point (Vq1) and 0.18 mV (empirical value), then Q2 is selected as the Q point. Otherwise, Vq1 and amplitude of Q2 (Vq2) are compared and the minimum point is considered the Q point. Similarly, the search space of 55 ms and 110 ms is considered after the R peak to determine the S point and minimum points locations are stored in the variables named S1 and S2. If S1 and S2 are similar then it is considered as the actual S point otherwise, the minimum point is considered as S point. A detailed discussion of finding out Q and S points is given in our earlier work [25].

### 3.2 Baseline and J point detection

**FIGURE 9:**
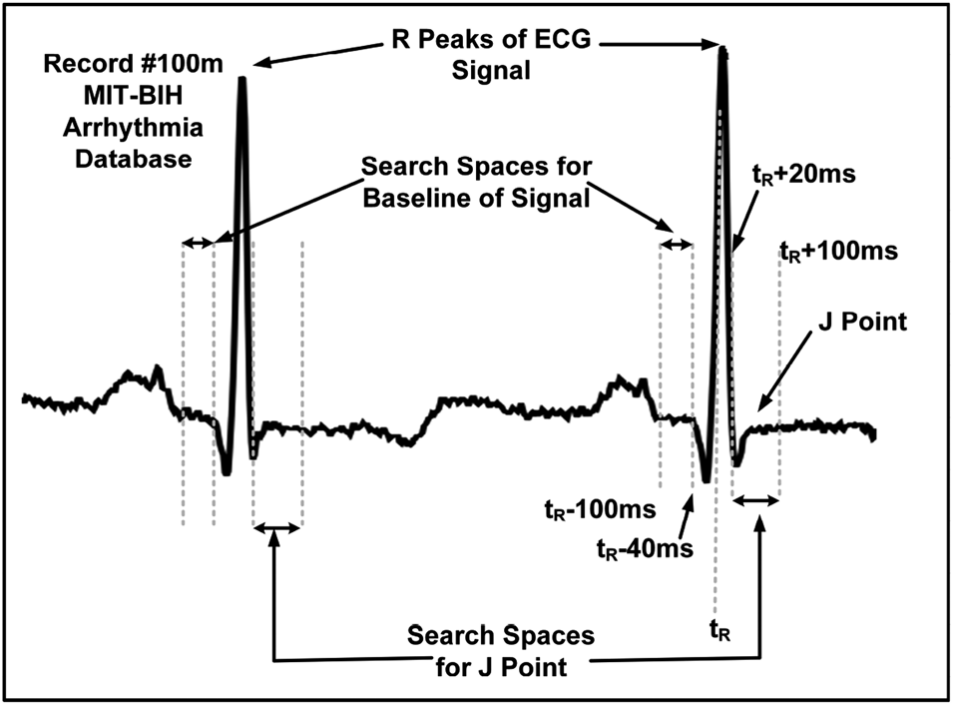
Search spaces for baseline (isoelectric level) and J point of the signal.

In our proposed algorithm, J point is detected in a search space of 80 ms starting from $t_{R}$ + 20 ms, where $t_{R}$ denotes the position of the corresponding R peak. We detect three successive points with a slope less than or equal to an empirical value (2.5μV/s). If the above mentioned condition holds true the midpoint of it has been considered as the J point. In the case where the condition is not satisfied, $t_{S}$ + 60 ms is considered as the J point [17], where $t_{S}$ represent the corresponding S point location. Local search spaces for baseline and J point is shown in Figure 9.

Additionally, for detecting the baseline of the ECG signal, a search space from $t_{R}$−100 ms to $t_{R}$−40 ms is considered. This particular segment is again searched for 20 ms duration with minimum average slope value. The selected segment's mean amplitude is defined as the baseline level or the isoelectric level of the signal. The J point and baseline are significant for cardiac anomalies.

### 3.3 Proposed T wave detection algorithm

**FIGURE 10:**
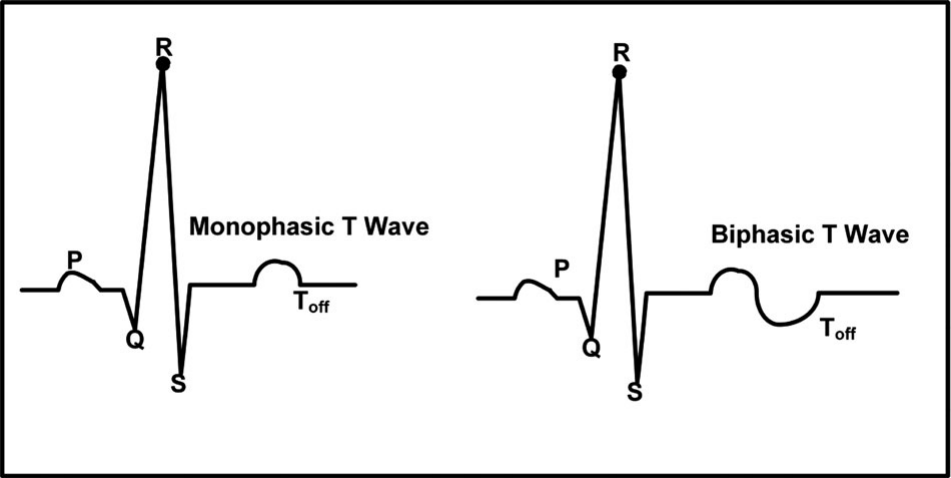
Monophasic and biphasic T waves.

For the detection of conditions like MI, hypocalcemia and hypercalcemia, T wave detection is essential. The nature of T wave can be monophasic or biphasic in different leads. As shown in Figure 10, the waveforms show the biphasic and monophasic nature of the T waves. Several T wave detection [26, 27] schemes are provided for the monophasic T waves.

**FIGURE 11:**
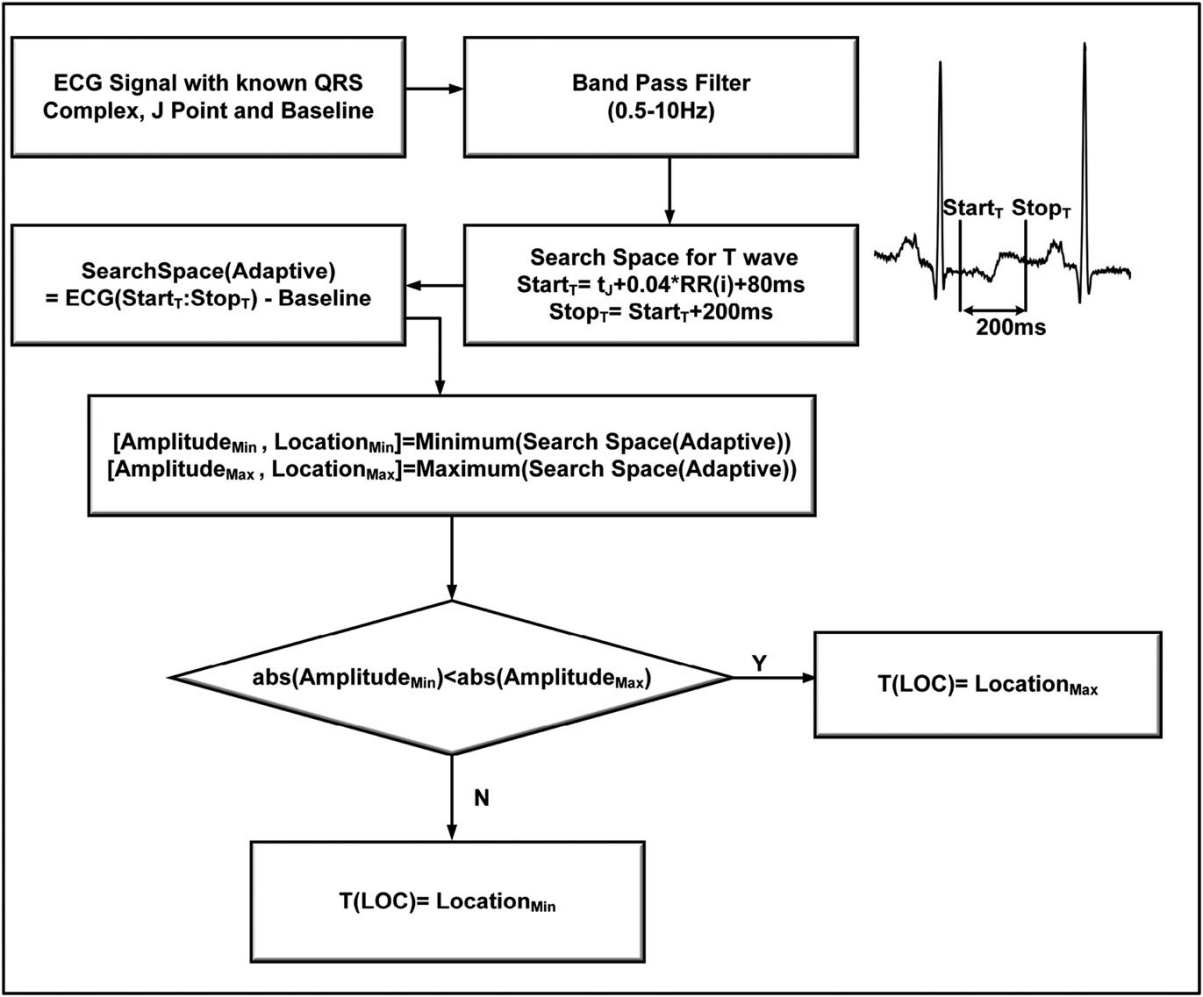
T wave detection for biphasic, monophasic, positive and inverted T waves.

To detect monophasic or biphasic T waves, our proposed algorithm utilizes the QRS complex, J point and baseline information of the signal. The corresponding RR intervals are also utilized to create reliable search spaces for the existing T waves. A 200 ms search space, as shown in Figure 11 is made adaptive to continuously occurring baseline changes or drifts in the signal, by subtracting the corresponding baseline level of the signal from the probable search space. It is to be noted that the 200 ms duration search space for possible T waves starts from the $Start_{T}$ point, where $t_{J}$ and RR(i) represent the J point location and corresponding RR interval, respectively.

For T wave detection, initially the ECG is passed through 0.5–10 Hz BPF to optimize the T wave energy at the output. Following this, the minimum and maximum amplitudes in the local search space and respective sample numbers (locations) have been determined. The biphasic or monophasic T wave location is then detected by comparing the absolute (abs) maximum and minimum values, as shown in Figure 11.

### 3.4 Proposed P wave detection algorithm

**FIGURE 12:**
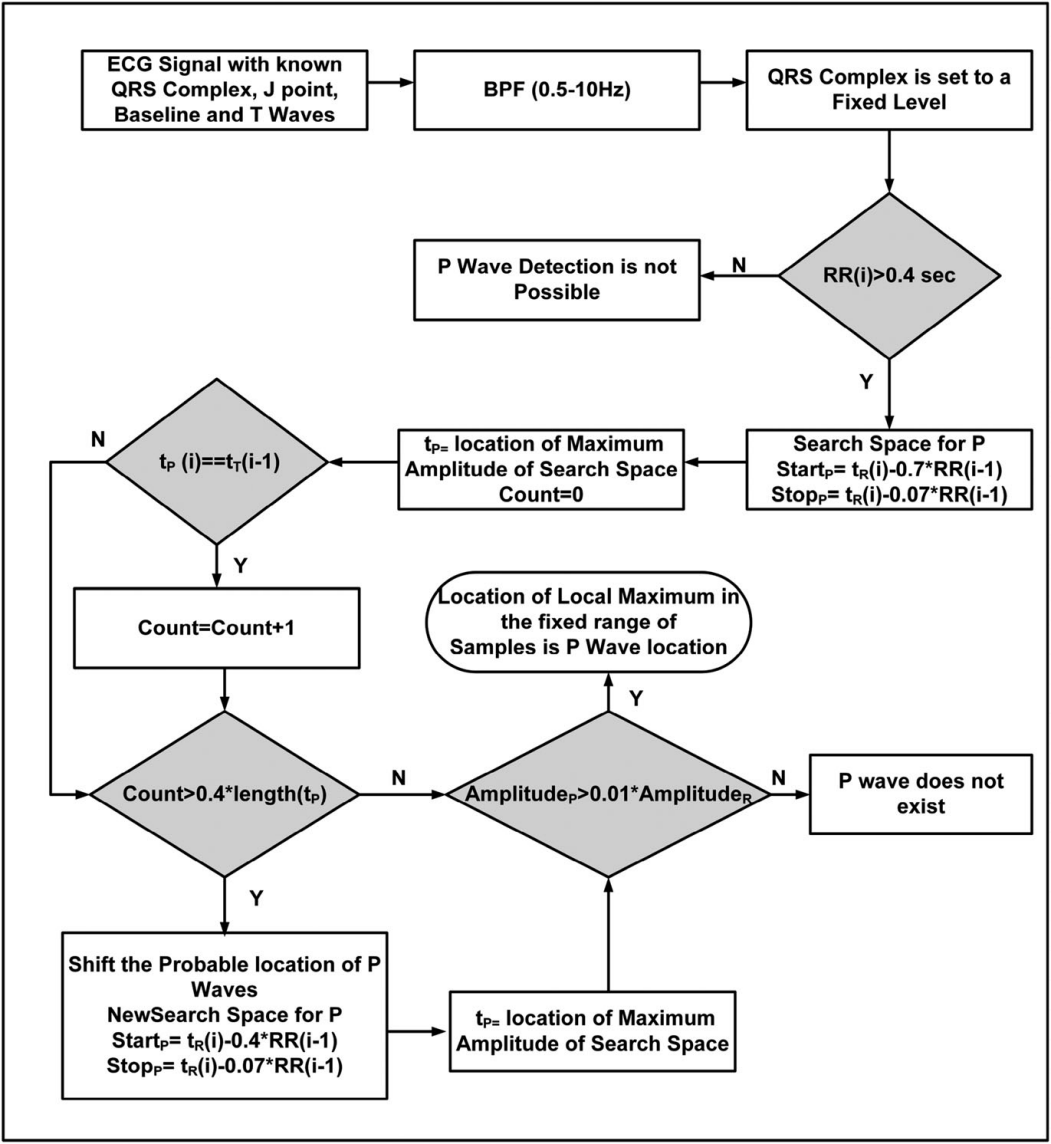
Proposed flow diagram for P wave detection.

P wave detection algorithm is proposed in this paper and the flow diagram is shown in Figure 12. In the beginning, the BPF optimizes the P wave energy at the output; the QRS complexes of the signal are set to fixed level so these do not obstruct the detection of P waves. For Premature Ventricular Contractions (PVC), the P waves may not occur due to certain physiological constraints [16]. To mitigate this, we have introduced a prerequisite for the P wave detection: if the corresponding RR interval of the signal is greater than a threshold value (0.4 s), we can only follow the remaining algorithm to detect the P waves. Otherwise, the P wave for that particular segment will not exist and hence cannot be detected.

The temporal search space for P wave mentioned in [28] is adopted in this work. The search space for the P wave starts from $Start_{P}$ and ends at $Stop_{P}$ (see Figure 12). The maximum amplitude in this search space is detected. The location of the maximum ($t_{P}$) in this region is compared with the previous cycle's T wave location ($t_{T}$). If the above mentioned condition is true for more than 40% (empirical value based on experimentation) times of the total length of T waves, it is concluded that T wave locations are misinterpreted as P wave locations and therefore the $Start_{P}$ is shifted to another location of the signal (see the flow diagram). By shifting the probable search spaces for the P waves in the cases of overlapping P and T wave locations, the proposed method can effectively distinguish between T waves and P waves in specific cases, such as broad PR and prolonged QT intervals etc.

Further, the location of maximum amplitude in this segment is considered the possible P wave location. Following this, P wave amplitude ($Amplitude_{P}$) is compared with 1% value of R peak amplitude ($Amplitude_{R}$), and if the condition holds true, a possible P wave is selected as the reasonable P wave. At the final stage, $t_{P}$ is again searched for the local maximum in the 40 ms region for obtaining the final P wave location.

## 4 ECG FEATURES AND DISEASE CATEGORIZATION

A detection sequence for determining the feature points R Peaks, QRS Complex, J Point, P wave and T wave is used as shown in Figure 6. These features are further used to diagnose various heart anomalies and disease categorization.

### 4.1 R‐R interval

**FIGURE 13:**
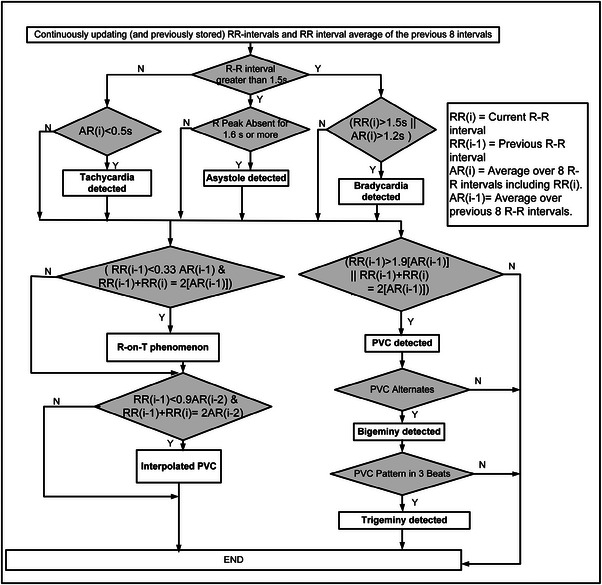
Detection algorithm for various arrhythmias.

For observing the various rhythmic changes, the R‐R intervals of lead II (connected between LL and RA) are preferred as this provides the best representation for these changes [16]. The detection of arrhythmias is based only on the rhythmic changes [29] associated with the signal. As shown in Figure 13 the algorithm detects tachycardia, asystole, bradycardia, R on T condition, bigeminy, trigeminy, PVC and interpolated PVC arrhythmias depending on the R‐R intervals. A detailed discussion is given in our earlier work [30].

### 4.2 ST‐T segment

The ST‐T segment anomalies are used to detect ischemic heart diseases or MIs [16, 31] that represent an insufficient supply of blood to the heart and missing it can be fatal. MI can be further subcategorized mainly as anterior, inferior, lateral and septal cases according to its effects on the various arteries connected with the heart. In conventional hospital settings, invasive methods such as blood troponin levels with 12 lead ECG signals are used to detect MI conditions. Our proposed system is aimed at ambulatory monitoring services that utilize the frontal leads for ST‐T deviated MI detection. The MI can cause morphological changes on the ST‐T segment of the ECG signal, such as Inverted T waves (IT), Hyperacute T waves (HT), ST Depression (STD) or ST Elevation (STE) above the baseline of the signal [16]. It is one of the observation that our proposed method take advantage of.

To detect HT, the R peaks amplitude is compared with T waves amplitude. If the amplitude of T peak is more than 80% of the corresponding R peak and it is comparable for most of the signal, then it is considered the HT case. Similarly, to detect the IT, the amplitude of J point and amplitude of T peak is considered for the comparison. If the J point amplitude is greater than the corresponding T peaks amplitude for most of the signal, it is considered the case of IT. To detect the STE and STD in the signal, we adopted the analysis from [32].

**FIGURE 14:**
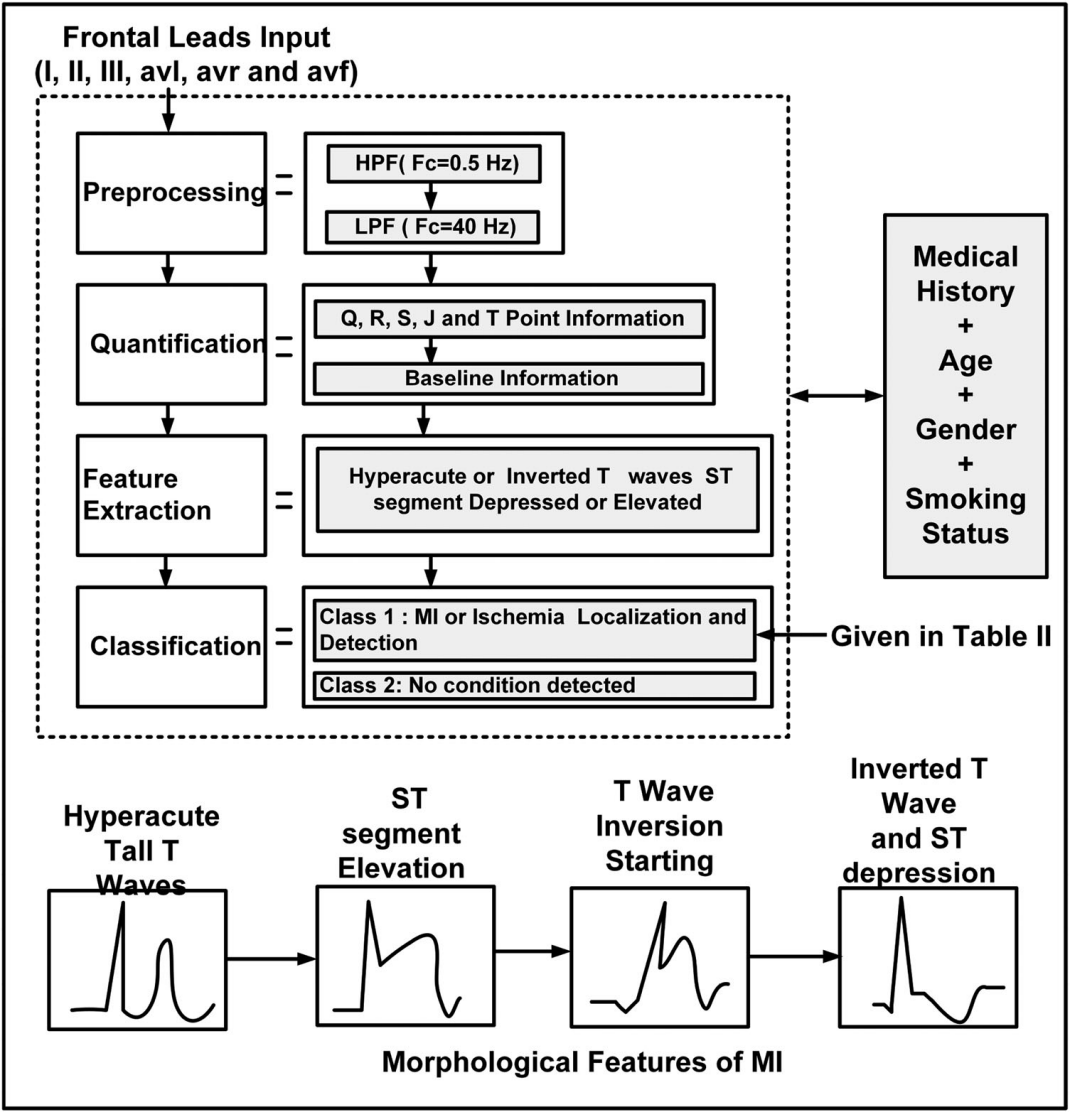
Detection algorithm for MI.

After detecting HT, IT, STE and STD in the contiguous leads, we considered novel feature user specific information (USI) such as gender, age and smoking status in the proposed algorithm. With the experimental results, we conclude that the inclusion of USI improve the algorithm efficacy, also mentioned in standard European Society of Cardiology [17, 18] where these features are vital in diagnosing the cardiac health of an individual. The MI cases are further subcategorized into lateral, inferior, anterior or septal and inferolateral cases. Morphological changes to localize MI into subcategories is shown in Table 1. The detection algorithm for MI with the morphological features is shown in Figure 14.

**TABLE 1:** Morphological features associated with various types of MI.

Types of MI	Morphological features
Inferior	**STE** (II, III and *avf*) AND **STD** (I and avl)
	OR **HT** (II, III and *avf*)
	OR **IT** (II, III and *avf*)
Lateral	**STE** (I and *avl*)AND **STD** (III and *avf*)
	OR **HT** (I and *avl*)
	OR **IT** (I and *avl*)
Inferolateral	**STE** (I, II, III and avl)
	OR **HT** (I, II, III and *avl*)
	OR **IT** (I, II, III and *avl*)
Anterior or Septal	**Injury pattern in** *avl* **[** **33** **]** OR
	**STD** (II, III and *avf*) [34]

In the standard 12 leads setup, anterior and septal MI detection requires the access of precordial or chest leads (V1, V2, V3, V4, V5 and V6). Still, in the proposed method, we have utilized the critical observations from [33] and [34] to use only frontal leads to detect anterior or septal MI cases. According to the observation, the injury pattern of *avl* lead (when STelevation>0.05mV or T wave Inversion) and ST depression in inferior leads (lead II, III and *avf*) are essential features for detecting anterior or septal MI cases. Based on our experiments, we conclude that utilization of *avl* lead and reciprocal leads led to the optimization of the system.

In our earlier research work [35], a comparison of single lead, 2 lead and 3 lead MI detection algorithms have been discussed in detail. It has been observed that 2 lead MI detection is best suited for resource constrained regions.

### 4.3 QT interval

QT interval is significant for detecting cardiac conditions such as hypocalcemia or hypercalcemia. It is the time interval between the starting of Q waves to the end of T waves ($T_{off}$). To detect the $T_{off}$ point, a duration of 80 ms has been considered starting from $T_{peak}+40$ ms where $T_{peak}$ represents the location of peak of T wave. In the above mentioned duration, a 16 ms segment with the minimum slope value has been determined by using the moving window operation. The starting point of the selected duration is considered as the $T_{off}$ point. To make the QT interval adaptive to RR interval variability, we utilized Bazett's formula and detected the corrected QT interval [16, 36] with the following formula:

(5) $$QT_{c}=\frac{QT}{\sqrt{RR}}$$

The normal range of $QT_{c}$ duration is between 390 ms and 460 ms. If the detected duration lies beyond the mentioned range, it signifies cardiac anomaly.

### 4.4 PR interval

The electrical signal passes from the atria to the ventricles of the heart through the AV node. The PR time delay occurs mainly due to the passage of the electrical impulse through the Atrioventricular (AV) node, which acts as a regulator of conduction. This corresponds to the PR interval that gives us information regarding the AV node anomalies. The boundary values for the PR interval are 120 ms and 200 ms, beyond this range the PR interval is considered as abnormal [16]. AV block and AV node anomalies can be detected with a PR interval of the ECG signal.

**FIGURE 15:**
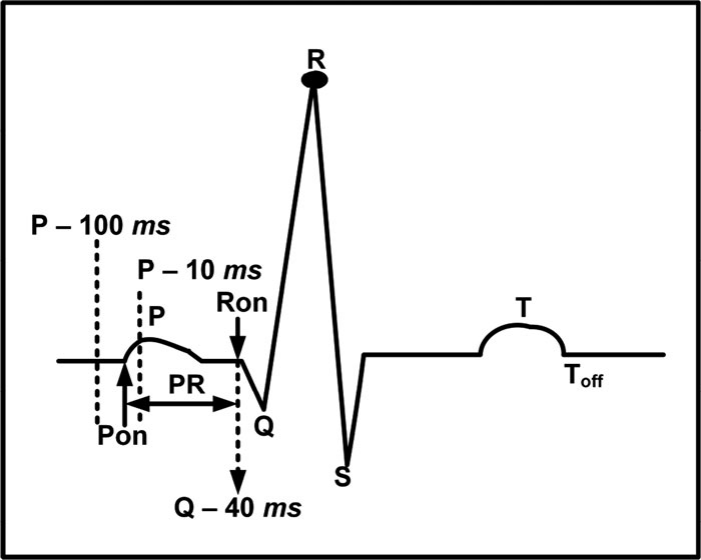
Search space for PR interval.

For determining the PR interval (see Figure 15), we require the $P_{on}$ and $R_{on}$ points on the ECG signal where $P_{on}$ represents the starting point of the P wave and $R_{on}$ is the starting point of QRS complex. For determining the $P_{on}$ we have determined the minimum slope interval of 16 ms duration within a search space of 100 ms to 10 ms prior to the P wave peak. The $R_{on}$ point is considered a fixed point 40 ms prior to the Q point.

### 4.5 P wave and R‐R interval

Often the absence of P waves and irregular R‐R interval signifies atrial fibrillation. Here, the atria do not provide the necessary impulses to the ventricles that cause the absence of P waves and irregular R‐R intervals. The R‐R information and P wave information is used to detect this condition. The features utilized for 30 mins of data are standard deviation of R‐R intervals and number of detected P waves in comparison with detected R waves while for 1 min of data, interquartile range of R‐R interval is used. The values of interquartile range and standard deviation are quite higher in the atrial fibrillation cases compared to the normal cases. Similarly, in atrial fibrillation cases, the length of detected P waves are at least 20% lesser than the length of detected R waves.

## 5 RESULTS AND DISCUSSIONS

This section discusses the results for feature points, disease detection and efficiency metrics on various databases.

To validate the proposed algorithms, we have utilized standard databases available on the Physionet [37] that demonstrate the robustness of our proposed method. The results obtained for automated ECG abnormality detection are invariably data dependent [23]. To make the algorithm comparable to the state of the art, MIT‐BIH Arrhythmia database [23] is utilized for the detection of QRS complex, the QT database [38] and MIT‐BIH arrhythmia database with P wave annotations [39] are utilized for the validation of the proposed T and P wave detection, respectively. The PTB database [9] and MIT‐BIH Arrhythmia databases are used for MI, atrial fibrillation and arrhythmia classification accuracy. Along with it, the field validation has been done with cases and controls in a Public Health Centre in Gujarat, India.

### 5.1 Description of databases utilized

For the validation of P, Q, R, S, T points and cardiac anomalies detection, we have utilized several databases available in the Physiobank [37]. A brief description of the databases that are used in this work is given as following.

#### 5.1.1 Physikalisch‐technische bundesanstalt database (PTB) [9]

The database contains 549 records from 290 subjects. Each record contains 15 simultaneously measured signals out of that 12 signals represent the standard 12 lead ECG signals and the remaining three signals are the Frank leads. This dataset consists of records for MI cases, other cardiac anomalies and normal cases which has been utilized for validating the MI detection algorithm. The sampling frequency of the database is 1 KHz.

#### 5.1.2 MIT‐BIH arrhythmia database (MITDB)[23]

This dataset contains 48 half‐hour recordings of two channel ambulatory ECG with a sampling frequency of 360 Hz for MLII and V5 lead signals. The database provides the R peak annotations for the validation of results. Further, the database consists of various arrhythmic test cases.

#### 5.1.3 QT database (QTDB)[38]

This database provides the PQRST boundaries for 105 fifteen‐minute recordings. Waveform boundaries are given for the subsets of recordings and manually determined by the annotators. At least 30 beats in each record were annotated. The sampling frequency for the database is 250 Hz and the dataset is an amalgam of various other MIT‐ BIH databases with the specific boundary conditions.

#### 5.1.4 MIT‐BIH arrhythmia P wave annotations (PWAVE)[39]

This database consists of 12 recordings with P wave annotations from the MITDB. The database is used to verify the P wave detection algorithm results.

#### 5.1.5 MIT‐BIH noise stress database (NSTDB) [40]

This database includes 12 half‐hour ECG recordings and 3 half‐hour recordings of noise typical in ambulatory ECG recordings. The database consists of different signal to noise ratios (SNR) data for the record #118 and #119 of MITDB. The data is provided with 24 dB, 18 dB, 12 dB, 6 dB, 0 dB and −6 dB SNRs. The dataset is utilized to verify the proposed P wave and T wave robustness over different SNRs.

### 5.2 Efficiency metrics

The efficiency for the ECG feature point detection and disease detection is typically measured in terms of True Positives (TP), False Positives (FP), False Negatives (FN) and True Negatives (TN) [17]. Here, TP result is the result that is correctly detected; False FP is a result that is detected but not present in the signal; FN when the condition or case is not detected but the presence of a particular condition is there in the annotations. TN is the result that is correctly undetected due to the nonexistence of the condition.

Based on the TP, FP, FN and TN values the efficiency metrics in Positive Predictivity (PPV), Sensitivity (SEN), Specificity (SP), False Detection Rate (FDR) and Accuracy (ACC) are calculated and are given as following.

(6) $$Positive\;Predictivity=\frac{TP}{TP+FP}$$

(7) $$Sensitivity=\frac{TP}{TP+FN}$$

(8) $$Specificity=\frac{TN}{FP+TN}$$

(9) $$False\;Detection\;Rate=\frac{FN+FP}{TP+FN}$$

(10) $$Accuracy=\frac{TP+TN}{TP+FP+FN+TN}$$

### 5.3 Feature point detection results

#### 5.3.1 QRS Complex detection

In our proposed algorithm, the QRS detection utilizes the PTA [4, 5] and difference operation method [24] on MITDB [23] and the results are compared with the given annotations. The results given in Table 2 signifies the overall QRS detection results for 24 h data available in database. The average FDR is found out to be 1.29% for the database. It signifies that the method detects most of the QRS complexes efficiently.

**TABLE 2:** Efficiency metrics for QRS complex detection.

Total annotated beats	Number of beats detected	Mean PPV%	Mean SEN%	Mean FDR%
**109864**	**109391**	**99.29**	**99.49**	**1.29**

#### 5.3.2 T wave detection

For the validation of the T wave detection algorithm, we have utilized the MITDB records from the QT database [38]. Total of 15 MITDB records are available in the QT database and it also provides the manual annotations for the normal beats. The results are compared with the manual annotations provided in the database. The proposed algorithm's overall SEN is found to be 97.78% as shown in Table 3. It is to be noted that QTDB only consists of normal beats annotations for the selected segments of the signal; therefore, the algorithm could not detect any FP outputs for the particular segments. Hence, the efficiency metrics for T wave detection is restricted to only SEN% values.

**TABLE 3:** Overall T wave peak and $T_{off}$ location detection evaluation.

Total annotated beats	TP	FN	SEN%
**673**	**658**	**15**	**97.78**

**FIGURE 16:**
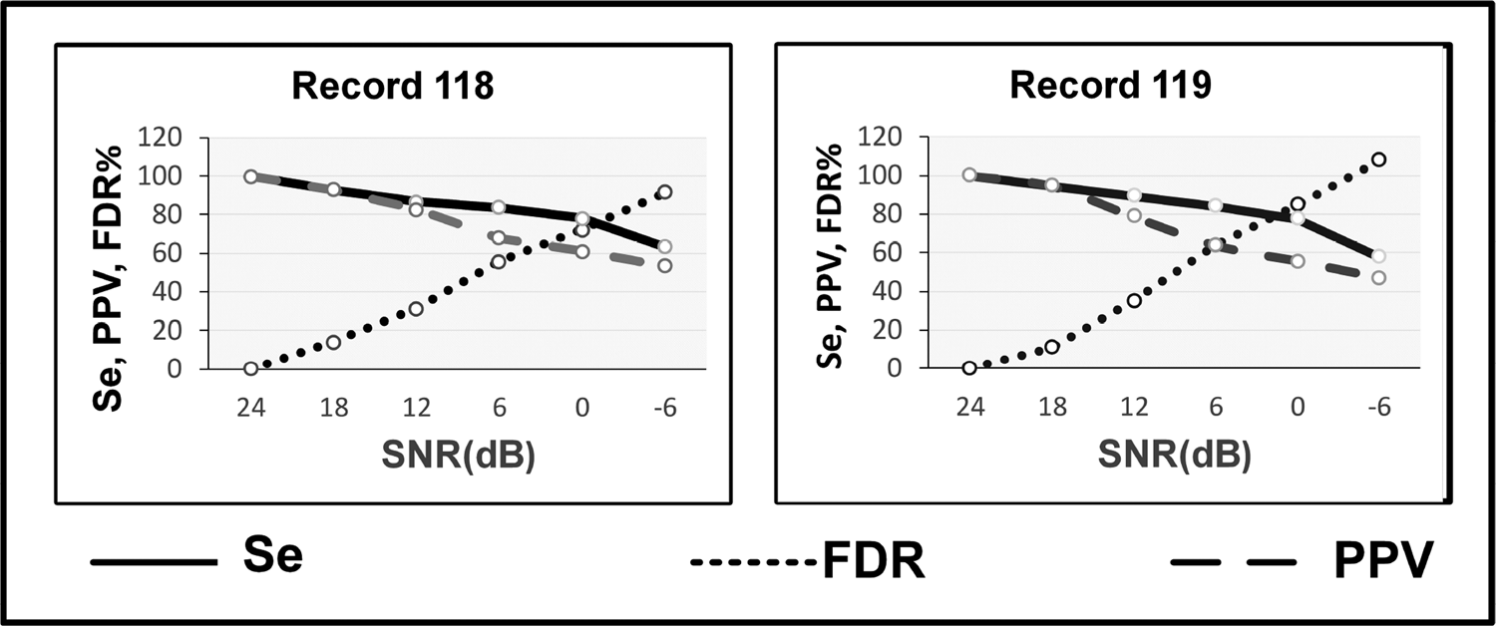
T wave noise stress analysis for two MITDB records.

Further, to check the robustness of the algorithm in a noisy environment, we have used the MIT‐BIH noise stress database. In this database, only record #118 and #119 are provided with different SNR values starting from 24 dB. We have assumed the result obtained for T waves on the 24 dB SNR to be ideal. We considered this to be the annotations for other data sets with varying SNRs from 18 dB, 12 dB, 6 dB, 0 dB and −6 dB to analyze the effects of noise. Figure16 shows the effects on SEN, PPV and FDR with the varying SNRs. A significant decrease in SEN , PPV and increment in FDR represents comparatively more false outputs with increase in noise values. Table 4 shows the state of art comparison of the proposed T wave detection algorithm. As can be seen in the table proposed method is comparable to the state of the art.

**TABLE 4:** State of art comparison for T Wave detection methods.

Algorithm	Method	Data used	SEN%	Limitations
[26]	Block of Interest	10 records	99.86	Not suitable for Biphasic T waves
[41]	Wavelet Transform	Selected beats	94.65	Trained on combined dataset
[42]	Multiscale Morphological Derivative	Selected segments	Ton= 99.8 Toff= 99.6	Not suitable for Biphasic T waves
**Proposed Method**	Temporal Local Search Spaces	15 Records from QTDB of MITDB Database	97.78	Requires QRS, J, Baseline information

#### 5.3.3 P wave detection

For validating the P wave detection algorithm, the MIT‐BIH arrhythmia database with P wave annotations is considered. This database contains 5 normal and 7 abnormal records with various cardiac anomalies. Records #100,#101,#103,#117 and #122 are the normal signals and Records #106,#119, #207, #214, #222, #223 and #231 are the abnormal signals with various arrhythmic conditions. For the normal and abnormal records, the overall FDR is 1.15% and 25.4%, respectively, that signifies the higher false results for the abnormal pathologies. The overall P wave detection results for the normal and abnormal signals is shown in Table 5.

**TABLE 5:** Proposed P wave detection algorithm validation results.

Records Category	TP	FP	FN	PPV%	SEN%	FDR%
Normal records	10141	58	60	99.43	99.4	1.15
Abnormal records	9866	2066	596	82.68	94.3	25.4

**FIGURE 17:**
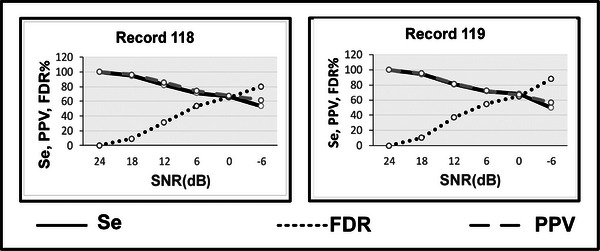
P wave noise stress analysis for two MITDB records.

Also, noise analysis is performed for P waves to verify the robustness of the algorithm on the noise stress database. An assumption is made that the obtained results at 24 dB are ideal and are compared as annotations for the remaining SNRs values ranging from 18 dB to −6 dB at an interval of 6 dB. Figure 17 shows the noise stress analysis for the P wave detection algorithm and it is concluded that decreasing SNR affects the results. The false outputs (FN, FP) also increase with the increase in noise levels, leading to higher FDR and lower SEN and PPV values.

The state of art comparison for proposed P wave detection is shown in Table 6. The proposed algorithm provides comparable results with various state of the art.

**TABLE 6:** State of art comparison for P wave detection methods.

Algorithm	Method	Data used	Signals	SEN%	PPV%
[28]	Phasor	MITDB P	Normal	98.42	99.98
	transform	Waves	Abnormal	96.40	85.84
[43]	Wavelet	MITDB P	Normal	77.84	75.03
	transform	Waves	Abnormal	96.26	76.74
[44]	Phasor	MITDB P	Normal	79.79	75.02
	transform	Waves	Abnormal	89.39	83.59
**Proposed**	Temporal	MITDB P	Normal	99.39	99.42
**Method**	search spaces	Waves	Abnormal	94.23	82.74

### 5.4 Disease detection results based on various features

The main morphological ECG features are utilized by our proposed algorithm to detect various cardiac conditions. The results obtained for the same are discussed following.

#### 5.4.1 R‐R features based arrhythmia detection

Various arrhythmic conditions [29] such as tachycardia, bradycardia, asystole and other such conditions are determined using R‐R features as shown in Figure 13. As shown in Table 7, the algorithm can detect most of the conditions. PVC and atrial premature contractions are the most common arrhythmias present in the MITDB.

**TABLE 7:** Results for arrhythmias detection.

Record	Arrhythmia present	Arrhythmia detected
100	PVC	PVC, Interpolated PVC
101	APC	PVC
102	PVC	PVC
106	Tachycardia, PVC, Bigeminy	Tachycardia, PVC, Trigeminy
108	Interpolated PVC, PVC	Interpolated PVC, PVC
114	PVC, Ventricular Couplets	PVC, Asystole, Bradycardia, Trigeminy
119	Trigeminy, Bigeminy	Trigeminy, PVC, Interpolated PVC
200	Tachycardia, PVC, Bigeminy, APC	PVC, Trigeminy, Tachycardia
201	Trigeminy	Trigeminy
203	Trigeminy, Tachycardia, Multiform PVC	Tachycardia, Trigeminy, PVC, Interpolated PVC
207	Bigeminy, Tachycardia	Tachycardia, Trigeminy, PVC, Interpolated PVC
214	Trigeminy, Tachycardia	Trigeminy, PVC, Tachycardia, Interpolated PVC, PVC
221	Tachycardia, PVC	Tachycardia, PVC, Trigeminy
222	Bigeminy	Tachycardia, PVC
223	Trigeminy, Tachycardia, Bigeminy, PVC	Tachycardia, Trigeminy, PVC, Interpolated PVC
232	Bradycardia, Asystole	Bradycardia, Asystole, PVC
234	Tachycardia, PVC	Tachycardia, PVC

#### 5.4.2 ST‐T segment anomalies based MI detection

To validate our algorithm, we have used the lead I and lead II signals from the 15 data present in the PTB database [9]. Our algorithm classifies and localize the MI cases and the efficiency metrics for the proposed algorithm with and without using USI is shown in Table 8. With the utilization of USI, the proposed 2 lead algorithm shows ≈10% improvement in accuracy compared to the case when it was not utilized. By applying weights to the user specific parameters, we achieved higher accuracy from the algorithm. For example, a male above 60 years of age and with positive smoking status has a higher probability of the disease compared to the counterpart [18]. Therefore, it is concluded that the inclusion of such parameters makes the algorithm robust for real‐time deployment.

**TABLE 8:** Efficiency metrics of proposed MI detection algorithm with & without USI.

USI	TP	TN	FP	FN	PPV%	SEN%	SP%	ACC%
✓	123	45	7	23	94.6	84.2	86.5	85
X	123	30	22	23	84.8	84.2	57.6	77.27

The algorithm detect and localize the site of infarct into three major categories. Inferior, anterior/septal and lateral MI has been detected based on all the four stages of MI. The results based on the site of infarcts are shown in Table 9, which signifies that even without precordial leads, the algorithm can detect most of the anterior MI cases.

**TABLE 9:** Types of MI cases in PTB and cases detected by proposed algorithm.

MI Categories	Subcategories	Total Cases	Detected Cases
Inferior	Inferior	36	30
	Inferolateral	25	19
	Inferoposterior	4	4
	Inferoposterolateral	9	6
Anterior	Anterior	23	21
	Anterolateral	18	17
	Anteroseptal	28	25
	Anteroseptolateral	1	1
	Anterior, Inferior	1	0
Lateral	‐	1	0

The state of the art comparison is shown in Table 10. In all the mentioned parameters in the table, our proposed method is comparable and in certain cases performs better compared to the state of art. Therefore, we conclude that the system offers itself as a useful screening tool for the post MI patients even at the home settings.

**TABLE 10:** Comparison of proposed MI detection algorithm with state of the arts.

Ref.	Method Utilized	Leads Config.	Dataset	SEN%	PPV%	SP%
[6]	Support vector machine	12 Lead ECG	PTB	94.6	_	96.0
[7]	Neural Network & Genetic Algorithm	12 Lead ECG	PTB	96.55	_	95.24
[8]	Morphological characteristics by support vector machine	12 Lead ECG	PTB	97.73	_	93.44
[11]	ECG & VCG indexes based Identification	VCG & 12 Lead ECG	PTB	95.8	‐	94.2
[45]	ST deviation detection based on morphological features	2 leads (Variable)	ST‐T	84	85	_
[46]	Morphological features for T deviations	2 leads (Variable)	ST‐T	83	75	_
[47]	QRS loop & parameter ST vector magnitude processing	VCG	Unknown	88.5	_	92.1
[48]	Wavelet transform based recurrence quantification analysis	VCG	PTB	96.5	_	75
**Proposed system**	**IT, HT, STE & STD Features of Frontal Leads with USI**	**Lead I, Lead II**	**PTB**	**84.2**	**94.6**	**86.5**

#### 5.4.3 QT interval validation

The validation of QT interval is verified with the $T_{off}$ point on QT database. The QT duration is the interval between the onset of QRS complex and $T_{off}$ point. The validation is restricted to the endpoint of T wave and QT duration beyond this as the QT anomalies (Hypocalcemia, Hypercalcemia etc.) are not provided in the database.

The 15 MITDB signals of QT database are considered for testing. Table 3 shows the results for Toff point detection as we have correctly identified the $T_{off}$ for the detected T peaks. Table 11 shows the corrected QT duration for the Record #Sel103m for the beats ranging from 2 to 12 while the first beat is not considered. We select the prolongation of QT interval as the feature for hypocalcaemia and shortening of QT interval, the feature for hypercalcaemia.

**TABLE 11:** $QT_{c}$ Duration for record Sel103 from QT database.

Beat no.	QRS Onset (Sample)	$T_{off}$ (Samples)	QT (Samples)	QT (sec)	R‐R (Samples)	R‐R (sec)	$QT_{c}$ (sec)
2	392	483	91	0.3640	216	0.864	0.3916
3	601	699	98	0.3920	209	0.836	0.4287
4	813	910	97	0.3880	211	0.844	0.4223
5	1022	1120	98	0.3920	210	0.840	0.4277
6	1238	1344	106	0.4240	217	0.868	0.4551
7	1470	1573	103	0.4120	231	0.924	0.4286
8	1690	1789	99	0.3960	219	0.876	0.4231
9	1890	1998	100	0.4000	209	0.836	0.4375
10	2106	2209	103	0.4120	208	0.832	0.4517
11	2317	2422	105	0.4200	210	0.840	0.4583
12	2525	2627	102	0.4080	209	0.836	0.4462

#### 5.4.4 PR interval validation results

The PR interval provides information regarding the AV node anomalies. The ideal PR interval must lie between 120 ms and 200 ms.

Otherwise, it signifies the AV node conduction anomalies such as AV nodal rhythm, AV block etc. The ground truth values for validating the PR interval is not provided in the database. Hence, we have calculated the PR interval for the initial few beats, for example, Record #Sel100 is considered and the results are shown in Table 12.

**TABLE 12:** PR duration for record Sel100 from QT database.

Beat no.	P Onset (Sample)	R onset (Samples)	PR (Samples)	PR (s)
3	476	524	48	0.192
4	662	707	45	0.18
5	856	897	41	0.164
6	1040	1085	45	0.18
7	1235	1283	48	0.192
8	1440	1488	48	0.192
9	1631	1684	53	0.212
10	1828	1877	49	0.196
11	2019	2065	46	0.184
12	2209	2258	49	0.196
13	2404	2446	42	0.168
14	2588	2632	44	0.176

#### 5.4.5 P wave R‐R features

Atrial fibrillation can be diagnosed by the absence of P waves and irregular R‐R intervals. For the 30 minute datasets, we have adopted standard deviations (S.D.) of R‐R interval and a number of detected P waves versus R waves to detect atrial fibrillation cases. However, for small duration datasets (≈1 min of data), these features do not provide the expected results as the standard deviation for a smaller range of data does not provide the required variability index for the R‐R intervals.

Table 13 shows some of the important features obtained for MITDB normal and atrial fibrillation affected waveforms for 30 min of data.

**TABLE 13:** Important features for atrial fibrillation detection for 30 min Data.

Waveforms	Record	Std. Deviation (R‐R)	# of R (waves)	# of P (waves)
Normal cases	100	17.48	2272	2268
	101	26.3	1868	1850
	103	17	2083	1935
	117	15.42	1530	1530
	122	15.11	2477	2471
Atrial fibrillation cases	201	135.58	1922	1373
	202	103.57	2127	1692
	203	82.058	2913	2123
	210	48.46	2614	2324
	217	33.79	2202	687
	219	82.46	2153	2084
	221	72.77	2424	1603
	222	83.16	2492	1983

To improve the detections on smaller datasets, the interquartile range (IQR) is considered to obtain the variations approximation. We have also observed that in the case of atrial fibrillation R‐R interval distribution is skewed instead of Gaussian. For determining the IQR, the RR interval is arranged in ascending order and determined by the following equations.

(11) IQR=Q3−Q1

(12) $$Q1\;Position=\frac{\left(length(RR)+1\right)}{4}$$

(13) $$Q3\;Position=\frac{(length(RR)+1)\times 3}{4}$$

**FIGURE 18:**
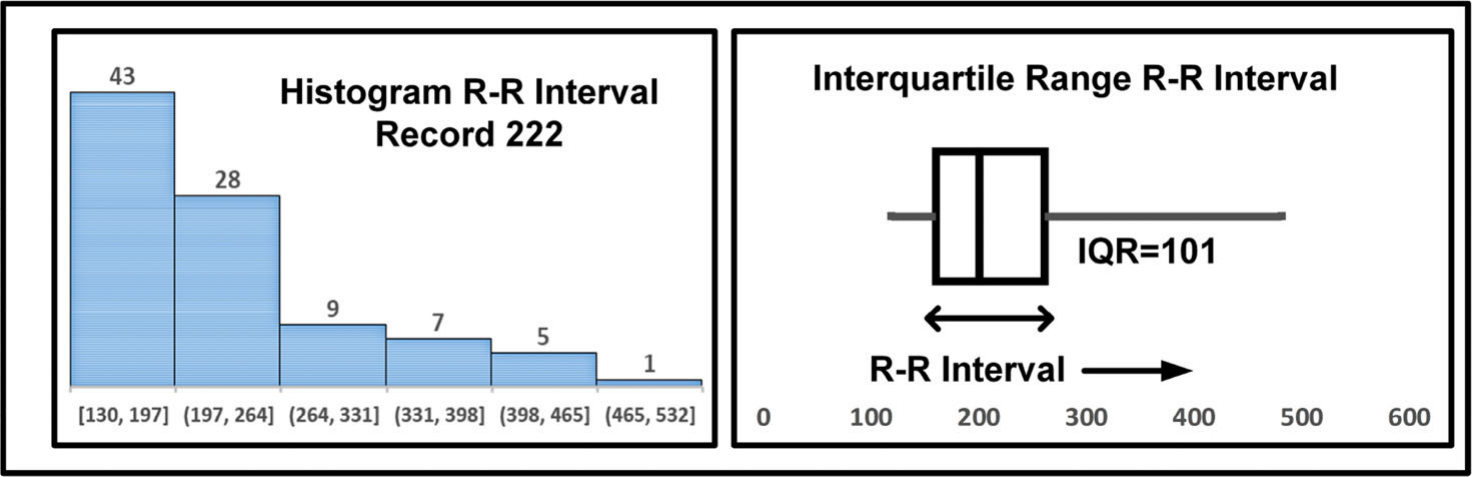
Histogram and box plot for R‐R interval record 222 MITDB.

Figure 18 shows the histogram and box plot of the IQR for the Record #222 of MITDB. It shows the data for 1 min interval leads to skewed distribution and hence, IQR (101) provides a better measurement for the variations.

**FIGURE 19:**
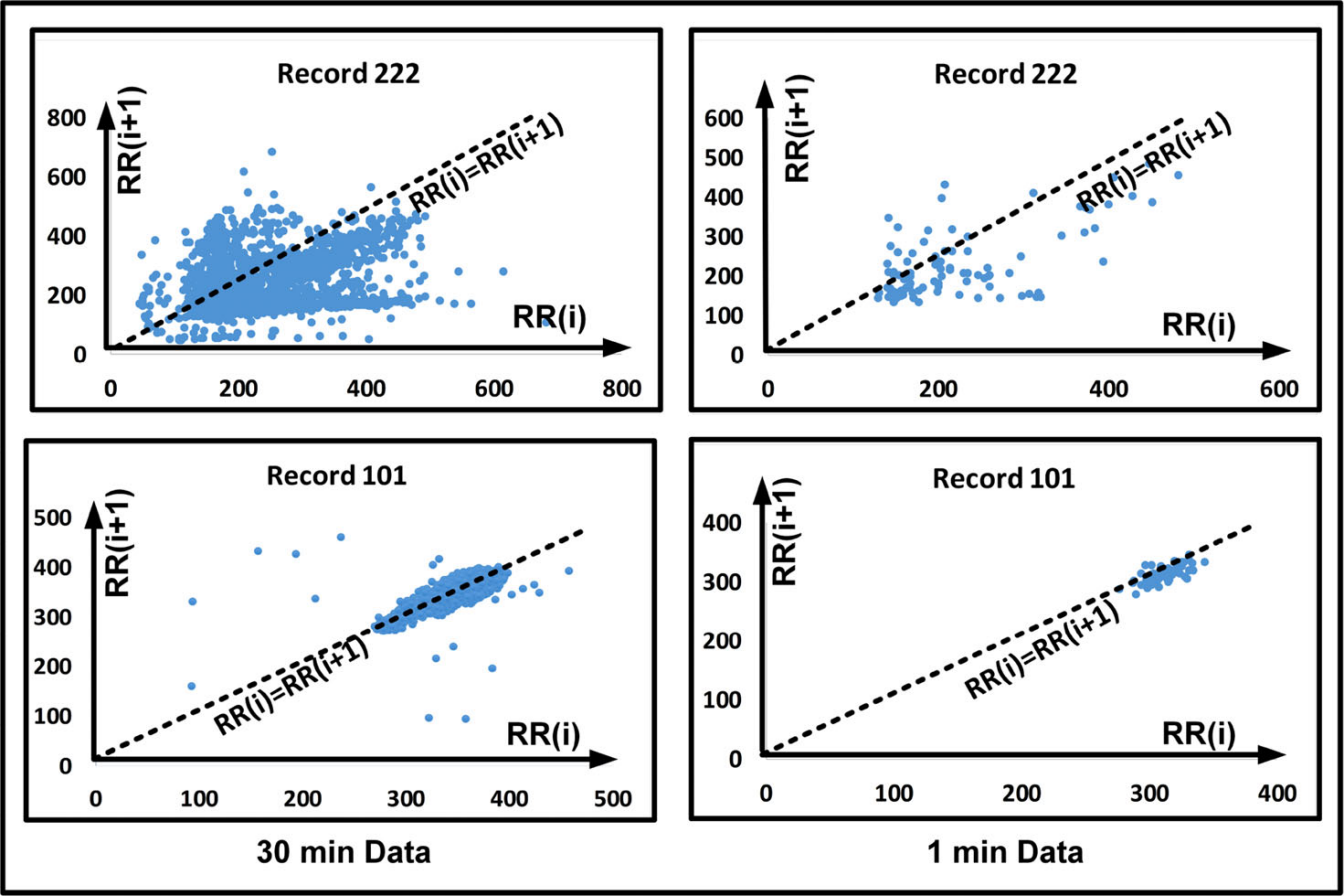
Poincare Plot for R‐R Interval for 1 min and 30 min data.

Figure 19 shows the Poincare plots for record #222 and record #101 for 30 min and 1 min data. It is to be noted that we have considered 1 min duration from the database with the atrial fibrillation episodes. As we can see the atrial fibrillation affected signals show more spread in Poincare plot as the R‐R interval is varying more compared to a normal waveform. Table 14 shows the IQR for normal cases and atrial fibrillation cases for 1 min of duration.

**TABLE 14:** Statistical IQR for atrial fibrillation detection for 1 min dataset.

ECG Cases	Record No.	IQR (R‐R)
Normal cases	100	27
	101	33.25
	103	37.75
	117	35.5
	122	23.25
Atrial fibrillation cases	201	79
	202	90.75
	203	279.25
	210	101.50
	217	50
	219	149
	221	155.75
	222	100.50

### 5.5 Field validation

Field visits have been performed in a Public Health Centre in Gujarat, India to validate the system in real‐time environment with cases and controls. In total 42, cases and controls have been considered for the validation. Out of these 42 cases and controls, 18 are TN, 15 are TP, 1 is FP, 8 are FN results The system provided the ACC of 78.5% for the total of 42 cases and controls. TP cases mainly consist of various types of MI and arrhythmias. It is to be noted that the automated results of the system were compared with the cardiologist's diagnosis on the 12 lead ECG data obtained in the hospital.

## 6 CONCLUSION

In this paper, cardiovascular atrial and ventricular disease detection front end along with the proposed algorithms for the back end has been demonstrated. Various critical features P wave, QRS complex and T wave of the ECG signal have been quantified in this work. R peaks, Q point and S point detection achieved PPV, SEN and FDR of 99.29%, 99.49% and 1.29%, respectively. The novel T wave detection method based on probable time domain spaces achieved the detection SEN of 97.78% for the MITDB signals available in the QTDB database. For the normal sinus rhythm signals, the proposed P wave detection algorithm achieved the overall PPV, SEN and FDR of 99.43%,99.4% and 1.15%, respectively. Similarly, for the abnormal pathologies, the values are 82.68%,94.3% and 25.4%. The work categorizes various diseases based on the R‐R segment, ST‐T segment, PR segment, QT segment, P wave and R‐R interval. Based on the mentioned features, detection for diseases such as arrhythmias, MIs, prolongation and shortening of QT and PR intervals, and atrial fibrillation has been done that corresponds to atrial and ventricular anomalies related to cardiac health. MI detection and localization obtains PPV, SEN, SP and ACC of 94.6%,84.2%,86.5% and 85% on the PTB database. Anterior and septal MI detection utilizes the injury pattern in *avl* lead and reciprocal changes in inferior leads as features and it makes the system sufficient by only using frontal leads. Atrial fibrillation detection ACC on MITDB is found out to be 100% for 30 min and 1 min data. It has been observed that the use of personal user information such as gender, age and tobacco usage records with the algorithm leads to more robust detections. The designed system for real‐time lead I and lead II signal acquisition and processing achieved 78.5% accuracy in detecting various cardiac anomalies therefore, we conclude that it is an ideal tool for improving cardiac health literacy in resource constrained regions.

## AUTHOR CONTRIBUTIONS

Neha Arora: Conceptualization, data curation, formal analysis, investigation, methodology, resources, software, validation, writing ‐ original draft, writing ‐ review and editing. Biswajit Mishra: Conceptualization, data curation, formal analysis, funding acquisition, investigation, methodology, project administration, resources, supervision, validation, writing ‐ review and editing.

## CONFLICT OF INTEREST STATEMENT

The authors have no conflicts of interest to declare.

## Data Availability

Some of the data is derived from public domain resources and the derived data is subject to third party restrictions.
